# Annotation-based feature extraction from sets of SBML models

**DOI:** 10.1186/s13326-015-0014-4

**Published:** 2015-04-15

**Authors:** Rebekka Alm, Dagmar Waltemath, Markus Wolfien, Olaf Wolkenhauer, Ron Henkel

**Affiliations:** Department of Multimedia Communication, University of Rostock, Joachim-Jungius-Str. 11, Rostock, 18051 Germany; Fraunhofer Institute for Computer Graphics Research IGD, Joachim-Jungius-Str. 11, Rostock, 18059 Germany; Department of Systems Biology and Bioinformatics, University of Rostock, Ulmenstr. 69, Rostock, 18051 Germany; Department of Mobile Multimedia Information Systems, University of Rostock, Albert-Einstein-Str. 22, Rostock, 18051 Germany; Stellenbosch Institute for Advanced Study (STIAS), Wallenberg Research Centre at Stellenbosch University, Stellenbosch, South Africa

**Keywords:** Feature extraction, Model similarity, Bio-ontologies, SBML

## Abstract

**Background:**

Model repositories such as BioModels Database provide computational models of biological systems for the scientific community. These models contain rich semantic annotations that link model entities to concepts in well-established bio-ontologies such as Gene Ontology. Consequently, thematically similar models are likely to share similar annotations. Based on this assumption, we argue that semantic annotations are a suitable tool to characterize sets of models. These characteristics improve model classification, allow to identify additional features for model retrieval tasks, and enable the comparison of sets of models.

**Results:**

In this paper we discuss four methods for annotation-based feature extraction from model sets. We tested all methods on sets of models in SBML format which were composed from BioModels Database. To characterize each of these sets, we analyzed and extracted concepts from three frequently used ontologies, namely Gene Ontology, ChEBI and SBO. We find that three out of the methods are suitable to determine characteristic features for arbitrary sets of models: The selected features vary depending on the underlying model set, and they are also specific to the chosen model set. We show that the identified features map on concepts that are higher up in the hierarchy of the ontologies than the concepts used for model annotations. Our analysis also reveals that the information content of concepts in ontologies and their usage for model annotation do not correlate.

**Conclusions:**

Annotation-based feature extraction enables the comparison of model sets, as opposed to existing methods for model-to-keyword comparison, or model-to-model comparison.

**Electronic supplementary material:**

The online version of this article (doi:10.1186/s13326-015-0014-4) contains supplementary material, which is available to authorized users.

## Introduction

Thanks to standardization efforts in Systems Biology [1], modelers today have access to high-quality, curated models in standard formats. The Systems Biology Markup Language (SBML) [2] is an XML-based standard format to encode models as interactions between biological entities. The emerging networks are furthermore enriched with semantic annotations [3] which link model parts to external knowledge in domain-specific ontologies (bio-ontologies) [4]. Many SBML models live in open model repositories such as BioModels Database [5], the Physiome Model Repository [6], or JWS Online [7]. These repositories distribute computational models and associated data in standard formats. They support necessary management tasks, including curation, annotation, search, version control, data visualization etc. to different extents.

BioModels Database implements a native, SQL-based search [5]. An alternative search is the *ranked model retrieval* [8]. Here, models and their annotations are mapped on pre-defined model features (e. g., model organism, author, biological entity), leading to a characteristic term vector for each model. The properties of this vector are numeric values mostly describing term frequency and inverse document frequency (TF-IDF) [9]. The ranking is determined by the comparison of search terms (i. e. provided keywords) with the extracted characteristic term vector per model. Current approaches are solely capable of comparing a set of keywords against an indexed corpus of models and retrieve matching models. In addition, it is possible to create a characteristic term vector directly from a model and, subsequently, query a corpus by example.

For example, a standard search for the keywords “cell cycle” in BioModels Database retrieves all models in the corpus that are relevant to the term “cell cycle”. Together, all models returned by this search can be seen as a new, cell cycle focused, model set (or corpus). The same is possible for keywords such as “apoptosis”, “calcium oscillation” or “NF- *κ*B”. At this point, we end up with different sets of thematically related models. To characterize such a set and, later on, compare them, features describing this specific model set will be helpful. However, it is problematic to identify suitable characteristics for arbitrary or thematically focused sets of models.

In this paper we present four methods for annotation-based feature extraction from arbitrary sets of SBML models. Our methods build on combinations of existing approaches for feature extraction [10-13]. We exemplify our methods by comparing the characteristic features of thematic sets to the features of arbitrary sets of SBML models. The thematic sets were extracted from BioModels Database and represent the cell cycle, apoptosis, calcium oscillation, and NF- *κ*B. Concepts, i. e. terms in the ontology, were extracted from three major bio-ontologies used to semantically enrich models (GO, ChEBI, SBO). We argue that our methods contribute to the determination of similarity between sets of SBML models. They also provide statistics on the use of ontology terms in SBML models, and on the relation between ontology terms and models.

## Background

### Bio-ontologies

SBML is an XML format. It uses an RDF scheme to add semantic annotations to model parts [14]. Among the ontologies that are used to enrich SBML models, we chose here the following three ontologies, which we believe are the most relevant in model annotation: An ontology of gene and gene product attributes, the *Gene Ontology* (GO) [15]; an ontology of chemical entities, the *Chemical Entities in BIology* (ChEBI) [16]; and an ontology for modeling in biology, the *Systems Biology Ontology* (SBO) [3].

The GO is proposed and maintained by the Gene Ontology Consortium. It aims at standardizing the representation of gene and gene product attributes across species and databases by a structured, precisely defined, common, controlled vocabulary. GO covers three domains. The most important relationships within each domain are is-a and part-of. Additionally, each concept is linked to other kinds of information, including many gene and protein keyword databases.

ChEBI is an ontology of chemical entities of biological interest. All database entries are is_a linked within the ontology. Chemical classifications of ChEBI are aligned with the classification of chemical processes in the GO, and the majority of chemical processes in GO are defined in terms of the ChEBI entities that participate in them.

The SBO provides a set of controlled vocabularies of terms commonly used in Systems Biology. It consists of seven orthogonal branches. Terms within each branch are linked by standard is_a relationships. Formal ties to SBO have been developed for several representation formats in Systems Biology. SBML elements^a^, for example, carry an optional sboTerm attribute, which allows for a precise definition of the meaning of encoded model entities and their relationships.

### Feature extraction from ontologies

For feature extraction it is important to group similar items and to find categories that represent the content of the objects.

Several techniques to determine similarity use distance measures as a basis. Common techniques are euclidian or cosinus distance in vector space [17] or the editing distance for text [9,17-19]. In the context of this work the techniques to distances in ontologies and tree structures are of significance.

The hierarchical structure of the ontology can be used to determine the (semantic) similarity between objects [17]. A distinction is made between two approaches; the graph-theoretic and information-theoretic approach.

Examples for the graph-theoretic approach are the works of Bernstein *et al.* [17] and Wang *et al.* [20]. They describe the traditional approach for distance determination in ontologies using the number of edges between the nodes. The inheritance structure is represented in a directed acyclic graph in which the specialization of objects increases with each level. In such a graph the ontology distance can be described as the shortest path between two nodes. The shorter the distance between two nodes, the more similar they are. The problem with this approach is the assumption that the edges represent uniform distances within a taxonomy; i.e. the semantic connections are of equal weight. Li *et al.* therefore investigate in [21] how path length, depth and local semantic density influence the quality of the similarity function. They come to the conclusion, that for a semantic knowledge base especially path length and depth are important to get similarity results that compare to the human perception of similarity. The similarity values are used in cluster analysis approaches for *hierarchical clustering* [22]. Applied to the feature extraction task, we group concepts based on their distance in the ontology graph for one bio-ontology at a time. The top-down approach starts with a cluster containing all concepts and then splits this cluster into smaller groups. The bottom-up approach starts with clusters only containing one concept. Those clusters are merged into larger clusters.

The most prominent representative of the information-theoretic approach is Resnik [12,13]. This approach exploits the *information content* of objects to compare. The more information two objects have in common, the more similar they are. The information content of a concept *c* is dependent on the concept’s probability. The probability *p*(*c*) is calculated by the frequency *freq*(*c*) of the concept and the count *N* of all concepts of the ontology. It is formally defined by Resnik [12]:

(1) $$ p(c) = \frac{freq(c)}{N}  $$

If all concepts in an ontology are subordinate to one item, then this item has the greatest probability of 1, because its classification always applies. However, the smaller the probability of a concept is, the higher is its information content. The information content *IC* can be calculated by the negative logarithm of the likelihood:

(2) $$ IC(c) = - \log_{2} p(c)  $$

For example, the root term of the Gene Ontology summarizes all concepts of the ontology and consequently has an information content of zero. A child concept such as establishment of localization (GO_0051234) that summarizes 1408 concepts has a higher information content of 3.34 and a leaf concept such as natural killer cell mediated cytotoxicity directed against tumor cell target (GO_0002420) has the highest information content of 10.59.

In order to determine the common information content of two objects, one considers the deepest element that classifies both objects together. The information content of this element is the degree of mutual information content.

The Information Content can be used to address the problem of overgeneralization when using parent concepts as representatives for child concepts [23]. The challenge of feature extraction in ontologies is to find summarizing features that do not generalize too strongly. Concepts further up in the ontology are less specific than concepts further down in the ontology and, thus, have less “information content”. Counting the number of references of a concept and its successor concepts would rank the general concept always highest, as it has more references. The counting approach does not consider the loss of specificity when moving up the ontology. Trißl *et al.* propose a similarity-based scoring function where a general concept must be supported by more references to yield a good score of representativeness.

For our work we identified the information-theoretic approach and especially the notion of the information content to be of interest. Furthermore, we considered existing approaches for feature extraction in other areas, such as text classification, and selected the document frequency to be to some extend applicable in extracting a pre-defined number of features from sets of SBML models.

The *document frequency* describes the number of documents in which a term occurs [10,11]. It is used to reduce a vocabulary by removing to rare or common words, respectively. In text classification, common words are removed, because they are not discriminating for any particular class. Rare words are eliminated because they are considered non-informative for category prediction and not influential in global performance. In our specific application, common concepts from bio-ontologies are kept because they are very convenient as features. The discriminating power of a concept is given by the feature value that is saved for each model. However, rarely used concepts are removed during the feature extraction process.

For example, the Gene Ontology Term mRNA catabolic process (GO_0006402) is referenced in over 40 documents, terms of the branch establishment of localization (GO_0051234) are contained in over 200 documents, while terms of cell killing (GO_0001906) are rarely annotated. While the first two terms could be suitable as features, cell killing is not suitable at all, because only a few annotated documents could be found by this term.

## Implementation

As a proof of concept, we implemented the four different methods described in Section “Results and discussion” in a prototype application^b^. We then tested all methods on seven different model sets, which we extracted from BioModels Database.

### Prototype

The prototype implementation incorporates two major technologies. First, ontologies are imported using the OWL API [24] and the JFact [25] reasoner. The Web Ontology Language (OWL) is a specification of the World Wide Web Consortium (W3C) to create, publish and to distribute ontologies based on a formal description language [26]. Most bio-ontologies are available in OWL format.

Second, all relevant information about the models and the ontologies is stored in a graph database [27]. A graph database is well suited for models in SBML structure and ontologies alike. It supports links between ontology concepts and SBML models, and it allows for efficient queries [28]. For evaluation purposes, we imported the ontology concepts and their taxonomic relationships and counted the number of annotations referring from a model to a particular ontology concept. The storage approach has been described in detail in an earlier publication [29].

### Test sets

We generated seven different test sets containing SBML models from BioModels Database [30]. Two model sets contain arbitrary models, four model sets have a certain biological focus, and one model set contains the complete BioModels Database (Additional file 1: Table S1).

The cell cycle set (CC) contains only models from the curated branch. This ensures ground truth in model annotation as annotations in the curated branch are manually reviewed [5]. In addition to the cell cycle set, the two random sets (RS1 and RS2), the thematic test sets for apoptosis (APOP), calcium oscillation (CA) and NF- *κ*B (NFKB), and the set containing all 490 curated models (BMDB) were assembled from the curated branch. In contrast to the CC set (containing 30 models) the thematic test sets APOP, CA and NFKB only contain about 13 models each.

Consequently, we rely on the cell cycle set in our analysis of methods, we use the three other thematic sets for evaluation purposes.

The models for all model sets were pre-selected using our previously developed retrieval algorithm [8]. For example, the first test set is a thematic set containing SBML encodings of published cell cycle models. We used the term “cell cycle” for a keyword based search to retrieve a list of relevant models. To exclude possible false positive search results we manually validated the retrieved models based on their reference publications, resulting in the 34 given models for cell cycle. The model sets APOP, CA and NFKB were compiled in the same way.

From the biological point of view, the test sets CC, APOP, and NFKB are thematically similar. NFKB, which is one of the most prominent transcription factors, is able to manipulate cyclins that drive the cell cycle [31] and additionally has stimulus dependent pro- or anti-apoptotic functions [32]. Moreover, the connection between cell cycle and apoptosis is presented by many cells starting their apoptotic cell fate decision from the cell cycle arrest (G1/S checkpoint), i. e. after caspase activation [33]. More recently, calcium oscillations were shown to influence NF- *κ*B activity depending on the calcium spike duration [34]. We deliberately introduced the NFKB set with strong relations to the CC and APOP sets to evaluate if our methods reflect these relations in terms of similarity of extracted features. The assumption is that biologically similar model sets share semantic annotations.

## Results and discussion

Our main hypothesis is that it should be possible to extract characteristic features from semantic annotations, both for thematic sets of models and for arbitrary ones. The following subsections explain our four methods for feature identification, based on the aforementioned feature extraction methods (Section “Implemented feature extraction methods”); discuss their applicability to feature extraction from model sets (Section “Applicability of methods”); show the distribution of model annotations in BioModels Database (Section “Distribution of SBO concepts in SBML models”); and discuss the results obtained from two selected methods when applied to the abovementioned test sets (Section “Feature extraction from arbitrary model sets”). We conclude that it is indeed possible to identify characteristic features. These features can, for example, help with model retrieval, comparison and clustering.

### Implemented feature extraction methods

Our methods are designed to identify a predefined, maximum number of features for each compiled set of models. All methods incorporate the structure of the underlying ontology when grouping the concepts within it. Parent concepts represent the group containing their child concepts. Consequently, the developed methods are only applicable to taxonomy-shaped ontologies. Method 1 depends only on the chosen ontology, but not on the input set of models. All other methods additionally consider the annotations in the given set of models.

**Method 1** is a top-down clustering. To decide on the suitability of a concept for characterization, the probability *p* of each concept in the ontology is determined, following Resnik’s definition (Equation 1). In the context of this work, the frequency *freq*(*c*) refers to the number of all concepts that are summarized by a parent concept *c*.

**Method 2** is a top-down clustering that considers both the ontology structure and the annotations used in models of the given set. Consequently, the real distribution of references to ontology concepts used in models is regarded. Selected features depend on the given set of models. For each concept in the ontology, we count the number of annotations that refer to it. We call this number entity frequency. Additionally, we store the sum of a concept’s entity frequency and its descendants’ entity frequencies as aggregated entity frequency *EF*. All concepts with *EF*>0 provide the basis for feature extraction. Method 2 re-uses the algorithm of Method 1. The algorithm is adjusted to the dynamic setting by using the entity frequency metric instead of the probability *p*(*c*). To better compare the balance of the branches, we will normalize *EF* as entity probability *ep*(*c*):

(3) $$ ep(c) = \frac{EF(c)}{EF(root)}   $$

**Method 3** is a bottom-up clustering relying on the same input as Method 2. It also uses the entity probability *ep*(*c*) but begins with the individual concepts, which are gradually merged to form greater clusters. The results of this method are nearly identical to the ones of Method 2, but the performance of Method 2 is much better.

**Method 4** is a bottom-up clustering that addresses the problem of overgeneralization. It uses an adaptation of the scoring function as described in [23]:

(4) $$ Score_{T}(c) = IC(c) \cdot EF(c)  $$

The *Score*_*T*_(*c*) for a grouping represented by the concept *c* considers the information content and the aggregated entity frequency. The information content is calculated depending on the probability of *c* (see Equations 1 and 2). A group is formed by merging concepts with the ancestor that reaches the highest possible score.

### Applicability of methods

We tested the applicability of all described methods on sets of SBML models taken from BioModels Database. Method 1 calculates the probability to hit a certain node in an ontology with a model entity. It condenses a given ontology to a defined number of features, based on the probability of a concept in the ontology only. Thus, the results obtained from Method 1 do not depend on the actual ontology concepts that are referenced in the model set. Consequently, it does not adapt to the specifics of the corpus under study. Therefore, Method 1 is only suitable to provide a static set of features, solely based on the underlying ontology. As a result we dismissed Method 1 for the problem of finding characteristics for arbitrary model sets. However, Method 1 calculates the distribution of concepts in bio-ontologies, as shown in Section “Distribution of SBO concepts in SBML models”. Method 2 and Method 3 rely on entity probabilities. Our evaluations show that Method 2 (top-down) and Method 3 (bottom-up) produce almost identical results. The direction is only relevant in the rare constellation that two concepts are subsumed to the same score. In the following, we consider Method 2 for further evaluations. Method 4 is a dynamic approach that calculates the score value by entity frequency and information content. Based on the unique scoring and the absence of splits, Method 4 generally finds fewer features than the prior methods. It also selects more specific features (located further down in the ontology tree) that are still representative for the model sets. In Section “Feature extraction from arbitrary model sets” we use Method 2 and Method 4 to discuss the specificity and distinctness of extracted features.

### Distribution of SBO concepts in SBML models

**Figure 1:**
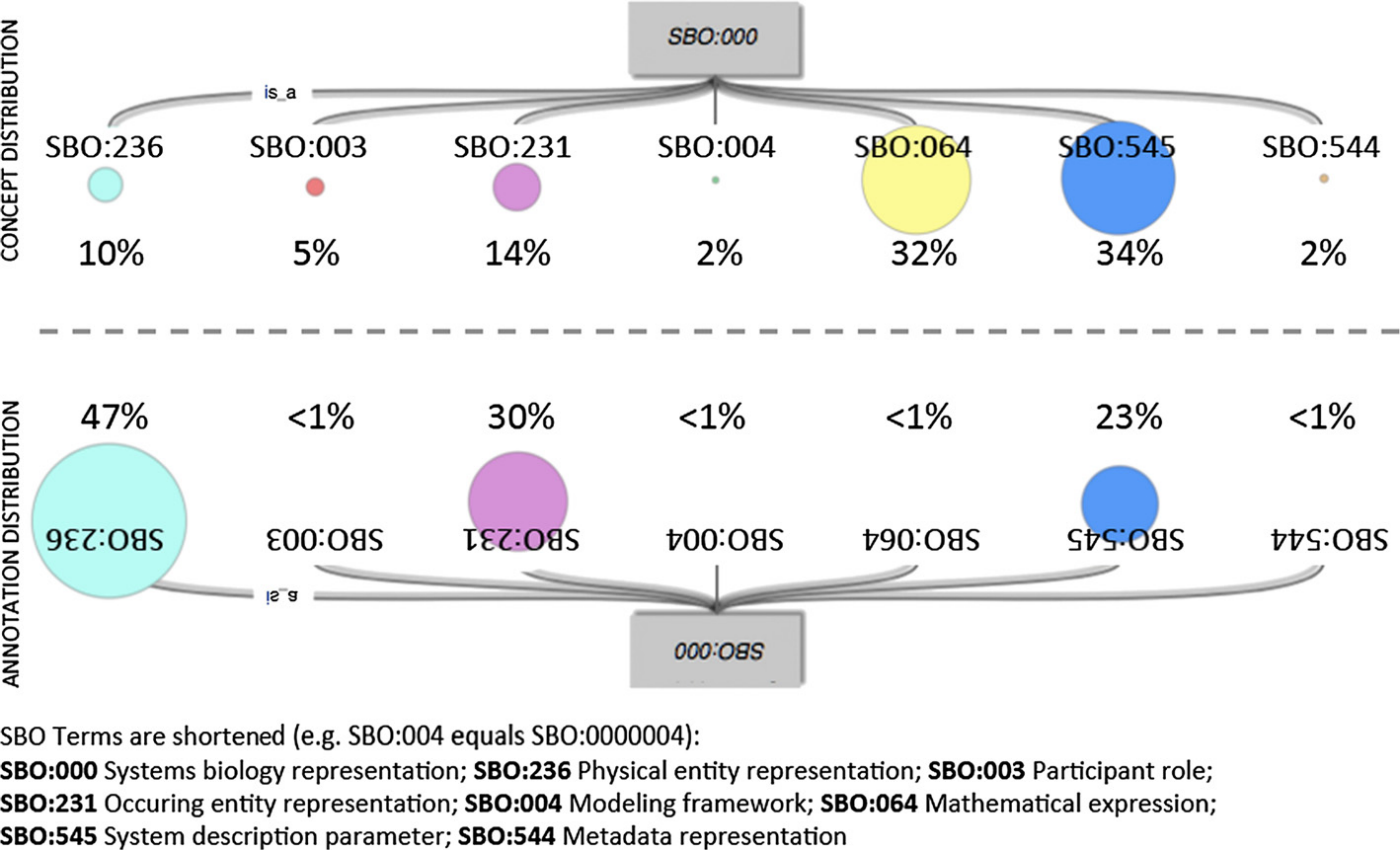
Concept vs. annotation distribution in SBO. Overview of the concept distribution in the seven branches of the Systems Biology Ontology (SBO). The size of the colored circles visualizes the number of concepts summarized by each branch. The bottom mirrored image visualizes the distribution of annotations from all models in the BioModels Database test set (BMDB). Figure adapted from [3].

Using Method 1, we compare the distributions of concepts in the SBO with the frequency of annotations as they occur in all models from BioModels Database. It becomes obvious that the concepts are unequally distributed across seven top-level branches (Figure 1, top). This is explained by the design of the SBO and its orthogonal branches. For example, the branch *modeling framework* (SBO:0000004) lists a “set of assumptions that underlay a mathematical description” whereas the branch *mathematical expression* (SBO:0000064) contains “formal representation of a calculus linking parameters and variables of a model”. Consequently, one expects more entries for *mathematical expression* than for *modeling framework*.

In conjunction with the application of SBO in model annotation, concepts of some branches are annotated more frequently (Figure 1, bottom). For example, the branch *physical entity representation* (SBO:0000236), which is a “representation of an entity that may participate in an interaction, a process or relationship of significance”, contains only 10% of SBO concepts, but 47% of the model annotations link to that branch. We expect that the characteristic features follow the distribution of the model annotations as seen in the lower part of the figure. Indeed, after applying Method 4, the selected SBO features show a distribution (66.6% physical entity representation (SBO:0000236), 6.6% participant role (SBO:00000003), 13.3% occurring entity representation (SBO:0000231), 6.6% mathematical expression (SBO:0000064), and 6.6% systems description parameter (SBO:0000545) that is closer to Figure 1 (bottom) than before; please refer to Table 1, Method 4, SBO, 15 features).

**Table 1:** **Extracted features for different sets (CC, RS1, RS2 and BMDB), methods and feature size**

**5 Features**	**Method 2**				**Method 4**			
	**CC**	**RS1**	**RS2**	**BMDB**	**CC**	**RS1**	**RS2**	**NFKB**
	33285	24870	24870	24870	22563	22563	26816	24870
	33302	33302	33302	33302	33608	26082	33695	26082
ChEBI	33304	33304	33304	33304	33694	33241	47019	33241
	35701	33582	33582	33582	37096	33695	61120	33695
	36357	36357	36357	36357	37787	61120	63367	61120
***avg depth***	5.4	4.2	4.4	4.2	7.2	5.6	8.2	5.4
	8152	3674	3674	3674	22411	3674	3674	3674
	9987	8152	5575	8152	30163	5575	9987	5575
GO	44699	9987	8152	9987	51726	6810	22607	9987
	65007	44699	9987	44699	65009	9987	43170	43170
	71840	51234	44699	65007	71822	43170	71822	71822
***avg depth***	2	1.8	1.8	1.8	4.4	2.2	3.6	2.6
	003	064	231	003	009	009	009	003
	236	231	245	064	231	064	167	009
SBO	374	240	247	231	252	176	240	064
	375	241	291	236	336	252		167
	545	545	545	545				240
***avg depth***	2.4	2.4	2.4	2	4	4.3	3.5	3
**15 Features**	**Method 2**				**Method 4**			
	**CC**	**RS1**	**RS2**	**BMDB**	**CC**	**RS1**	**RS2**	**BMDB**
	16646	18059	18059	18059	22563	22563	24875	24835
	24651	24835	24835	24835	33608	24835	25107	24870
	25367	24870	24870	24870	33694	25741	26816	26082
	25699	25367	25367	25367	37096	26082	33252	33241
	25741	25806	26082	26082	37787	33241	33620	33259
	26082	26082	33259	33241		33252	33636	33636
	33241	26835	33304	33259		33259	33695	33695
ChEBI	33839	33241	33581	33285		33608	35155	35155
	35701	33259	33674	33304		33695	35569	35569
	36358	33285	33839	33674		35701	47019	35701
	36606	33674	35701	33839		61120	61120	47019
	51143	33694	37577	35701		63367	63161	61120
	63161	35701	50906	50906		64709	63367	63161
	63299	51143	51143	51143				63367
	64709	64709	64709	64709				64709
***avg depth***	5.9	5.3	4.8	4.8	7.2	5.4	7.0	6.3
	3674	3674	3674	3674	216	3674	3674	3674
	5575	5575	5575	5575	4693	5575	5834	5575
	6807	6807	6807	8152	5575	6810	6826	9987
	9056	9056	9056	9987	22411	9987	8943	43170
	9058	9058	9058	32501	30163	16088	9987	71822
	40007	44237	32501	32502	32268	43170	22607	
	44237	44238	44237	40007	45750	45750	43170	
GO	44238	44699	44238	44699	51726		71822	
	44699	44710	44699	48511	65009			
	50896	48511	44710	50896	71822			
	51234	50896	50896	51234				
	65007	51234	51234	51704				
	71704	65007	65007	65007				
	71840	71704	71704	71840				
		71840	71840					
***avg depth***	2.3	2.3	1.9	1.8	4.1	2.1	3.0	2.6
	009	064	016	003	009	009	009	003
	177	177	017	064	231	064	167	009
	179	179	046	241	252	176	240	064
	180	180	153	245	336	252		167
	181	182	156	247				240
	182	185	231	253				
	205	205	241	285				
SBO	245	241	245	290				
	253	247	247	291				
	290	250	253	374				
	291	253	290	375				
	308	285	291	405				
	342	290	308	409				
	360	377	360	412				
	374	545	380	545				
***avg depth***	4.6	4.5	3.7	3.3	4	4.3	3.5	3

The upper table shows a maximum of five features, the bottom table 15 features, respectively. IDs are shortened (e.g. SBO:0000064 is represented by 064) and ordered ascending. The average depth (*avg*) of features per ontology is emphasized for the test sets.

**Figure 2:**
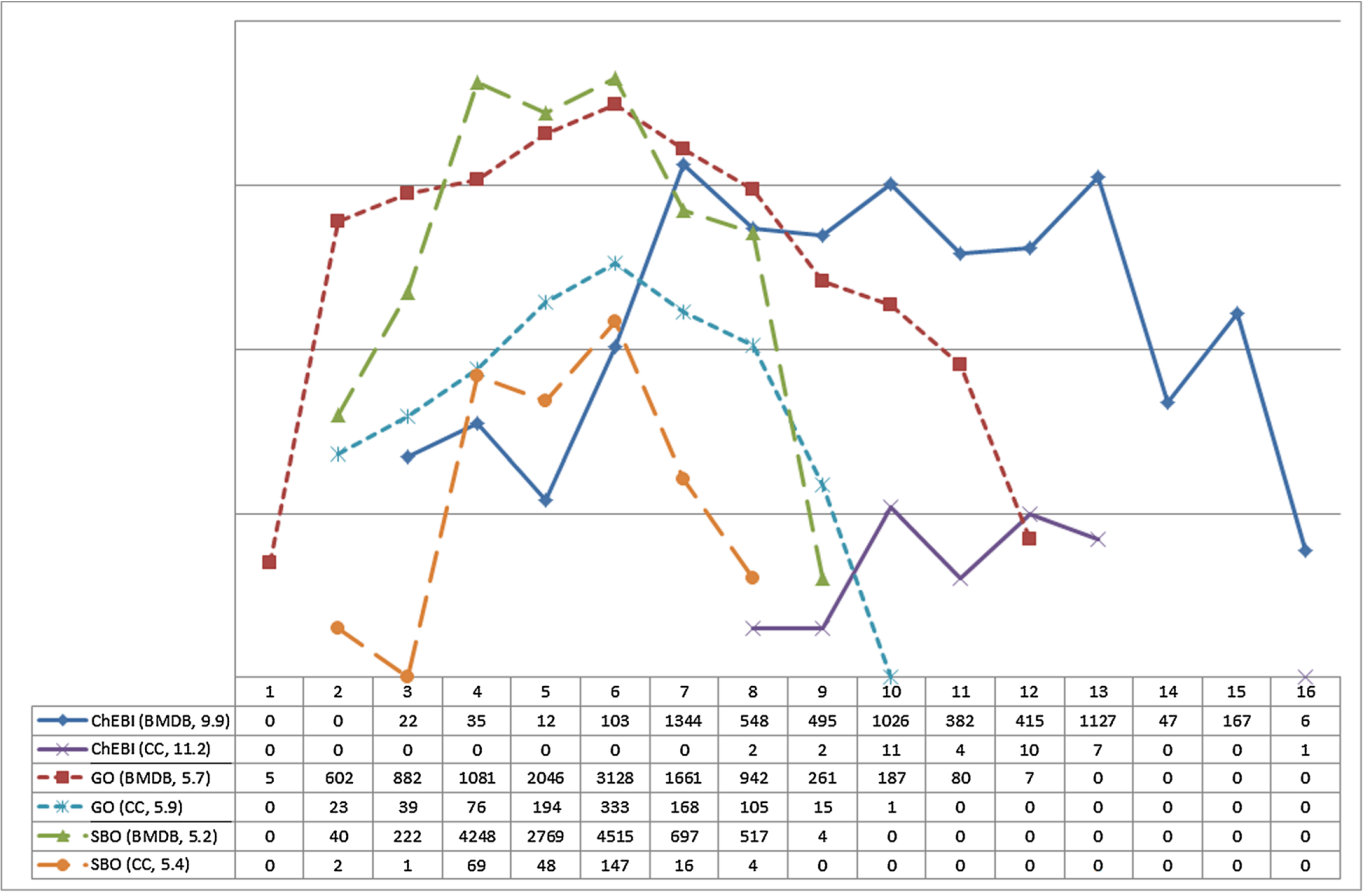
Concept depth of annotations. Distribution of annotation depth. Overview of the distribution of annotated model entities in relation to the depth of the annotation. The x-axis shows the depth of the annotated concepts in the corresponding ontology, the y-axis shows the number of annotated entities on a logarithmic scale (exact values are stated at the bottom of the figure). The figure legend states the ontology name, the model set and the average depth.

We also investigated for each model set the distribution of the depth of annotated concepts in the ontology tree. This knowledge helps us to decide on how specific a model annotation is. Figure 2 shows the distribution for model annotations using ChEBI, GO and SBO (Additional file 2).

Here, we plotted the distribution of annotations for the *CC* and the *BMDB* sets. As one would expect, both test sets show normal distributions. The 30 models contained in the *CC* set make up 6% of the 490 models in the *BMDB* set. However, the number of annotations in the *CC* set that refer to ChEBI is less than 1% compared to the number of annotations in the *BMDB* set. It should be considered that very sparsely annotated model set may be inferior in terms of specificity and distinctness. This information helps us later on in Section “Feature extraction from arbitrary model sets” to decide on the value of the extracted features.

### Feature extraction from arbitrary model sets

We hypothesize that the vast property space of a set of models can be condensed into a smaller, but still descriptive, number of features. To establish such “characteristic features”, we collect the models’ annotations and analyze the semantics behind the linked ontology terms. We focus on the semantics behind the model elements because we believe that this information will be most influential. All our methods require setting a maximum number of features.

Here, we chose to run our extraction methods with five and 15 features as an upper limit. The resulting sets of features for all feature extraction algorithms, models, and ontologies are shown in Table 1.

**Specificity of selected ontology concepts.** Table 1 shows the average depth of concepts in all three ontologies for all identified features in the *CC* and *BMDB* sets. Additionally, Figure 2 contains the average depth of annotation for the *CC* and *BMDB* sets before applying the feature extraction methods. The data confirms that the average depth of annotations decreases for Methods 2 and 4 (for all three ontologies and both model sets). Thus, selected concepts are higher up in the ontology, and more generic. This behavior is expected as the feature extraction process also involves generalization. However, the features extracted by Method 4 are more specific than the features extracted by Method 2. This is in accordance with the design of Method 4 to prevent overgeneralization. Moreover, the average annotation depth for the *CC* set is higher than for the corresponding *BMDB* set. This supports our assumption that thematically similar models share more annotations, and consequently the extracted features are more specific. For example, the concepts that were selected from ChEBI by Method 2 with a maximum of 15 features for the *CC* set have an average annotation depth of 5.9. In contrast, the concepts that were selected for the *BMDB* set only have an average depth of 4.8. According to our obtained data we infer that Method 4, in general, provides features that correspond to deeper concepts in the ontology than the features obtained from Method 2. We conclude from our test data that the depth of chosen concepts decreases with the increased randomness in the sets of models. This is not unexpected, as a broader data basis should not be characterizable by very specific ontology concepts. Rather, an arbitrary model set should cover many different semantic concepts, leading to more generic features being extracted. This behavior is also reflected in our data. In summary, both methods extract features that are specific to the model set. However, features extracted by Method 4 are mostly more specific than those extracted by Method 2. An exemption where the average depth slightly increases is Method 4 for SBO and 15 features. SBO is relatively small compared to GO or ChEBI. As Method 2 is required to select 15 features and Method 4 is only required to select up to 15 features, Method 4 selects only the most relevant features whereas Method 2 selects exactly 15 features. Due to the size of SBO, Method 2 adds features that are not best matches, nevertheless have a higher depth within SBO. This phenomenon did not occur for the lager ontologies, GO and ChEBI.

**Figure 3:**
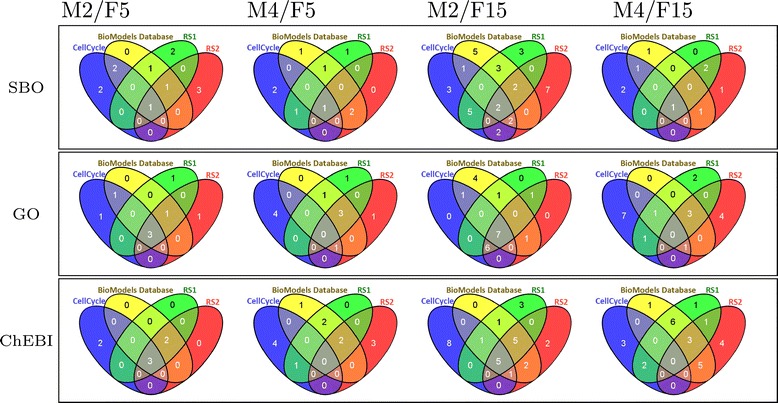
Feature overlaps. Visualization of feature overlaps of the four test sets. Each diagram shows the overlap of the results of one ontology (SBO, GO or ChEBI), method (M2 or M4) and number of features (F5 or F15).

**Distinctness of feature sets.** Another important question is how distinct the obtained features are for our test sets. If the methods retrieved similar concepts for the four test sets, then the extracted features could not be regarded specific to the set of models. Consequently, we measure overlap of concepts between the different characteristic features that we calculated with Method 2 and Method 4. Ideally, there would be almost no overlap of features selected for the *CC* set with any other selected set, whereas an overlap between *BMDB* and the random sets is expectable. Our results are shown in Figure 3.

A good result is achieved for Method 4 using 15 features and GO. Here, the cell cycle features have almost no overlap. The result achieved for Method 2 using 15 features and GO is not satisfiable. Here, the cell cycle features largely overlap with at least two other sets. However, the Venn diagrams, in general, confirm that both methods determine features that are specific to the model sets. They contain higher numbers of overlapping features at the intersection between arbitrary sets and very few overlapping features at the intersection between the *CC* and the *BMDB* sets. This is particularly visible for the results obtained from Method 4.

**Similarity of model sets.** We are also interested in how characteristic the sets of extracted features are for a given set of models. We first calculate the similarity of two concepts within the same ontology, as described by Li *et al.* [21]:

(5) $$ S(c_{1}, c_{2}) = \mathrm{e}^{- \alpha l} \cdot {{\mathrm{e}^{\beta h} - \mathrm{e}^{- \beta h}} \over {\mathrm{e}^{\beta h} + \mathrm{e}^{- \beta h} }}  $$

The variable *h* is the depth of the least common subsumer of the concepts *c*_1_ and *c*_2_, and the variable *l* is the length of the shortest path between both concepts. Following [21], the parameters are set to *α*=0.2 and *β*=0.6. We calculate this similarity value for each possible combination of features from two sets of models.

Afterwards we apply an adaptation of the Hungarian method [35] to the matrix resulting from the above calculations. The Hungarian method, a solution for the assignment problem, aligns pairs of features, in a way that ensures a global maximum similarity. Based on this similarity of features, we then calculate the total similarity of two sets of features, which corresponds to the similarity of the associated sets of models. The results are shown in Table 2.

**Table 2:** **Similarity between thematic and arbitrary model sets, calculated based on the similarity of their characteristic features**

**Model sets**	**Ontology**	**Method/number of features**			
		**M2 F5**	**M4 F5**	**M2 F15**	**M4 F15**
BMDB & CC	ChEBI	0.82	0.57	0.75	0.20
	GO	0.80	0.40	0.71	0.30
	SBO	0.75	0.44	0.50	0.43
BMDB & RS1	ChEBI	1.00	0.94	0.91	0.71
	GO	0.87	0.84	0.67	0.59
	SBO	0.75	0.65	0.63	0.65
CC & RS1	ChEBI	0.82	0.63	0.77	0.29
	GO	0.67	0.25	0.90	0.36
	SBO	0.50	0.63	0.70	0.63

Firstly, we discuss specificity of extracted features for the cell cycle set versus the set containing all curated models from Biomodels Database, and one random set. Desirable are low similarities for *BMDB vs CC* as well as *CC vs RS1*. As *CC* is a thematic set, its extracted features should differ from the features extracted from the *BMDB* and arbitrary model sets. A higher similarity is expected for *BMDB vs RS1*, as both sets represent a wide range of model topics. The results in Table 2 reflect our expectations. Particularly, the similarity values for Method 4 using 15 features clearly distinguish the extracted features of two sets. Method 2 using five features still shows the desired result, but due to the limited number of features the selected ones are more general and not very distinguishable. Even though results of Method 2 show the expected behavior, we conclude that the results of Method 4 are superior.

Secondly, we discuss the specificity for all thematic sets. Here, we narrow our scope to the Gene Ontology. As Table 3 indicates only the number of distinct annotations using GO is sufficient for all four thematic sets. In addition, we manually reviewed the extracted features and deduced that the features extracted for GO have the highest biological meaning. We use the aforementioned approach to calculate similarity between extracted features of six sets (BMDB, RS1, CC, APOP, CA, NFKB), as shown in Tables 1 and 4. Results for five and 15 selected sets are shown in Table 5. It becomes obvious that the similarities for Method 2 are to high in general, this supports our previous assumption of Method 2 over-generalizing the extracted features. An example of over-generalization is Method 2 using 5 features and the sets RS2 and NFKB. Both sets perfectly match. The reason for this match is, that Method 2 selected only top and second level representatives (both sets have an average depth of 1.8).

**Table 3:** **Number of curated model contained in each thematic data set**

	**Models**	**SBO**	**GO**	**ChEBI**
BMDB	490	13012	10882	5729
CC	34	227	954	37
CA	13	6	62	9
APOP	13	31	43	3
NFKB	12	28	35	0

Additionally, the number of distinct annotations contained in a set are shown for SBO, GO and ChEBI.

**Table 4:** **Extracted features for thematic test sets, methods and feature size**

**5 Features**	**Method 2**				**Method 4**			
	**CC**	**APOP**	**CA**	**NFKB**	**CC**	**APOP**	**CA**	**NFKB**
	8152	3674	3674	3674	22411	5515	5217	5515
	9987	5575	9987	8152	30163	30693	5829	6886
GO	44699	8152	44699	9987	51726	44257	6816	22607
	65007	9987	51234	44699	65009	65003	15085	44257
	71840	71840	65007	71840	71822	71822	51480	71822
***avg depth***	2.0	1.6	1.8	1.8	4.4	4.2	8.2	4.6
**15 Features**	**Method 2**				**Method 4**			
	**CC**	**APOP**	**CA**	**NFKB**	**CC**	**APOP**	**CA**	**NFKB**
	3674	3824	3824	3674	216	2090	5217	5515
	5575	5488	4872	5575	4693	5575	5783	5634
	6807	5575	5215	6807	5575	16265	5829	6886
	9056	9056	5488	9056	22411	30693	6816	16563
	9058	9987	5575	9058	30163	31264	15085	22607
	40007	30234	7204	44237	32268	43027	17111	44257
	44237	32501	22411	44238	45750	44257	38023	71822
GO	44238	44238	32469	44699	51726	65003	51480	
	44699	44699	44237	44710	65009	71822		
	50896	50896	50789	50896	71822			
	51234	51234	51234	51234				
	65007	65007	51481	65007				
	71704	71704	51716	71704				
	71840	71840	60089	71840				
			65009					
***avg depth***	2.2	2.1	4.0	2.4	4.1	4.2	7.0	4.3

The upper table shows a maximum of five features, the bottom table a maximum of 15 features, respectively. IDs are shortened (e.g. GO:00003674 is represented by 3674) and ordered ascending (Additional file 3).

**Table 5:** **Similarity between two model sets, calculated based on the similarity of their characteristic GO features**

**5 Features**	**BMDB**	**RS1**	**RS2**	**CC**	**APOP**	**CA**	**NFKB**
BMDB		0.8395	0.4720	0.3989	0.3522	0.0747	0.3629
RS1	0.8720		0.3203	0.2472	0.1917	0.1072	0.2746
RS2	0.8720	0.8720		0.5752	0.4078	0.1332	0.4632
CC	0.8000	0.6720	0.8000		0.4669	0.1116	0.5222
APOP	0.6720	0.6720	0.8000	0.6000		0.0912	0.7550
CA	0.8720	0.8720	0.7440	0.6720	0.5440		0.1758
NFKB	0.8720	0.8720	1.0000	0.8000	0.8000	0.7440	
**15 Features**	**BMDB**	**RS1**	**RS2**	**CC**	**APOP**	**CA**	**NFKB**
BMDB		0.5997	0.4800	0.2995	0.2016	0.0467	0.2592
RS1	0.6706		0.4230	0.3596	0.1573	0.0536	0.2476
RS2	0.9543	0.6706		0.3236	0.3202	0.0833	0.4105
CC	0.7185	0.8907	0.6727		0.3711	0.0811	0.3080
APOP	0.6449	0.6533	0.6449	0.7000		0.1543	0.5082
CA	0.3095	0.3364	0.3095	0.3315	0.4679		0.2022
NFKB	0.6681	0.9333	0.6681	0.9496	0.6953	0.3291	

Values for M4 are shown above the main diagonal, M2 below, respectively.

Desirable are low similarities for each thematic set versus the BMDB, RS1 or RS2 set, respectively. Both result tables show according similarity values for Method 4. We expect NFKB to have a slightly higher similarity to the other three thematic sets as NF- *κ*B has a regulatory effect on cell cycle, apoptosis and calcium oscillation. For five and 15 selected features Method 4 fits our expectation. The relation between CC and APOP is also visible as many cells start apoptosis from the cell cycle arrest. This is also supported by Method 4 for five and 15 features, respectively. In contrast, we predict CA to be distinct from CC and APOP as calcium oscillation has low overlap with cell cycle or apoptosis. Again, Method 4 advocates our prediction. In conclusion, Method 4 was able to support all our assumptions, even if only five characteristic features are provided per set.

## Conclusions

This paper presents and discusses methods for the annotation-based extraction of characteristic features from sets of SBML models. The methods consider clustering and text classification techniques to extract characterizing features for sets of annotated computational models in biology. Annotation-based feature extraction enables the comparison of sets of models, as opposed to existing methods for model-to-keyword comparison, or model-to-model comparison.

We evaluated four different methods for feature extraction and conclude that Method 4 is the most suitable. This method considers both, the semantic annotations in a set of models, and the information content of the ontology concepts. For our seven test sets, we showed that the extracted features are specific and distinct. In addition, we demonstrated that the extracted features are not overgeneralized. Thus, our expectations have been met: A thematic set of models, for example cell cycle models, can computationally be distinguished from arbitrary and other thematic sets of models. Finally, we suggested how to assign a similarity value to sets of models, based on the similarity of the extracted features.

Our applied methods are format agnostic and expandable. They can be adapted to other model representation formats such as CellML [36] or NeuroML [37]. Interestingly, these extensions enable a comparison between sets of models of arbitrary formats. It is also possible to incorporate further bio-ontologies, e. g. BRENDA [38].

For the near future, we plan to integrate Method 4 in our system for ranked model retrieval [8]. We wish to test the implications of feature extraction on model comparison and, in particular, model retrieval. We will also incorporate a larger set of ontologies into our system and ultimately in the process of feature extraction.

## Endnotes

^a^ Since Level 2 Version 2.

^b^ our code repository is available at https://bitbucket.org/ronhenkel/masymos.

## Additional files

Additional file 1 **Supplementary material.** A landscape table. This table lists for each of our seven test sets the contained models by their Biomodels Database ID.

Additional file 2 **Depth of ontology entries.** This file lists the number of annotations pointing to a certain depth within an ontology for each model set.

Additional file 3 **Extracted features.** This file lists extracted features and corresponding depth for each model set, feature size and ontology.

## References

[1] Hucka M, Nickerson DP, Bader GD, Bergmann FT, Cooper J, Demir E (2015). Promoting coordinated development of community-based information standards for modeling in biology: the COMBINE initiative. Frontiers in bioengineering and biotechnology.

[2] Hucka M, Finney A, Sauro HM, Bolouri H, Doyle JC, Kitano H (2003). The systems biology markup language (SBML): a medium for representation and exchange of biochemical network models. Bioinformatics.

[3] Courtot M, Juty N, Knüpfer C, Waltemath D, Zhukova A, Dräger A (2011). Controlled vocabularies and semantics in systems biology. Molecular systems biology.

[4] Robinson PN, Bauer S (2011). Introduction to Bio-ontologies.

[5] Li C, Donizelli M, Rodriguez N, Dharuri H, Endler L, Chelliah V (2010). BioModels Database: An enhanced, curated and annotated resource for published quantitative kinetic models. BMC systems biology.

[6] Yu T, Lloyd CM, Nickerson DP, Cooling MT, Miller AK, Garny A (2011). The physiome model repository 2. Bioinformatics.

[7] Olivier BG, Snoep JL (2004). Web-based kinetic modelling using jws online. Bioinformatics.

[8] Henkel R, Endler L, Peters A, Le Novère N, Waltemath D (2010). Ranked retrieval of Computational Biology models. BMC Bioinformatics.

[9] Baeza-Yates R, Ribeiro-Neto B (1999). Modern Information Retrieval.

[10] Yang Y, Pedersen JO (1997). A Comparative Study on Feature Selection in Text Categorization. Proceedings of the Fourteenth International Conference on Machine Learning. ICML ’97.

[11] Forman G (2003). An extensive empirical study of feature selection metrics for text classification. The Journal of machine learning research.

[12] Resnik P (1995). Using information content to evaluate semantic similarity in a taxonomy. Proceedings of the 14th International Joint Conference on Artificial Intelligence.

[13] Resnik P (1999). Semantic similarity in a taxonomy: An information-based measure and its application to problems of ambiguity in natural language. Journal of Artificial Intelligence Research.

[14] Waltemath D, Swainston N, Lister AL, Bergmann F, Henkel R, Hoops S, et al. SBML Level 3 Package Proposal: Annot. available online, Nature Preceedings. 2011. http://precedings.nature.com/documents/5610/version/1

[15] Ashburner M, Ball CA, Blake JA, Botstein D, Butler H, Cherry JM (2000). Gene Ontology: tool for the unification of biology. Nature Genetics.

[16] Hastings J, de Matos P, Dekker A, Ennis M, Harsha B, Kale N (2013). The ChEBI reference database and ontology for biologically relevant chemistry: enhancements for 2013. Nucleic acids research.

[17] Bernstein A, Kaufmann E, Bürki C, Klein M, Ferstl OK, Sinz EJ, Eckert S, Isselhorst T (2005). How Similar Is It? Towards Personalized Similarity Measures in Ontologies. Proceedings of 7. Internationale Tagung Wirtschaftsinformatik.

[18] Algergawy A, Nayak R, Saake G (2010). Element similarity measures in xml schema matching. Inform Sci.

[19] Schallehn E, Sattler K-U, Saake G (2004). Efficient similarity-based operations for data integration. Data Knowl Eng.

[20] Wang BB, Mckay RI, Abbass HA, Barlow M (2003). A comparative study for domain ontology guided feature extraction. Proceedings of the 26th Australasian Computer Science Conference - Volume 16. ACSC ’03.

[21] Li Y, Bandar ZA, McLean D (2003). An approach for measuring semantic similarity between words using multiple information sources. Transactions on Knowledge and Data Engineering.

[22] Hastie T, Tibshirani R, Friedman J (2009). Hierarchical Clustering. The elements of statistical learning.

[23] Trißl S, Hussels P, Leser U, Bodenreider O, Rance B (2012). InterOnto – Ranking Inter-Ontology Links. Data Integration in the Life Sciences. Lecture Notes in Computer Science, vol. 7348.

[24] Horridge M, Bechhofer S (2011). The owl api: A java api for owl ontologies. Semantic Web.

[25] JFact DL Reasoner: http://jfact.sourceforge.net/

[26] McGuinness DL, Van Harmelen F (2004). Owl web ontology language overview. W3C recommendation.

[27] Henkel R, Wolkenhauer O, Walthemath D (2015). Combining computational models, semantic annotations and simulation experiments in a graph database. Database.

[28] Waltemath D, Wolkenhauer O, Le Novère N, Dumontier M. Possibilities for integrating model-related data in computational biology. In: CEUR Workshop Proceedings of the 9th International Conference on Data Integration in the Life Sciences: 2013. Available online, www2.unb.ca/csas/data/ws/dils2013/

[29] Henkel R, Le Novère N, Wolkenhauer O, Waltemath D (2012). Considerations of graph-based concepts to manage computational biology models and associated simulations. GI-Jahrestagung.

[30] Release 25 of BioModels Database: ftp://ftp.ebi.ac.uk/pub/databases/biomodels/releases/2013-06-18/BioModels_Database-r25_pub-sbml_files.tar.bz2

[31] Joyce D, Albanese C, Steer J, Fu M, Bouzahzah B, Pestell RG (2001). NF- *κ*B and cell-cycle regulation: the cyclin connection. Cytokine Growth Factor Rev.

[32] Kaltschmidt B, Kaltschmidt C, Hofmann TG, Hehner SP, Dröge W, Schmitz ML (2000). The pro- or anti-apoptotic function of NF- *κ*B is determined by the nature of the apoptotic stimulus. Eur J Biochem.

[33] Pucci B, Kasten M, Giordano A (2000). Cell cycle and apoptosis. Neoplasia.

[34] Zhu L, Song S, Pi Y, Yu Y, She W, Ye H (2011). Cumulated Ca2+ spike duration underlies ca2+ oscillation frequency-regulated NF *κ*B transcriptional activity. J Cell Sci.

[35] Kuhn HW (1955). The hungarian method for the assignment problem. Naval research logistics quarterly.

[36] Cuellar AA, Lloyd CM, Nielsen PF, Bullivant DP, Nickerson DP, Hunter PJ (2003). An overview of CellML 1.1, a biological model description language. Simulation.

[37] Gleeson P, Crook S, Cannon RC, Hines ML, Billings GO, Farinella M (2010). NeuroML: a language for describing data driven models of neurons and networks with a high degree of biological detail. PLoS computational biology.

[38] Schomburg I, Chang A, Placzek S, Söhngen C, Rother M, Lang M (2013). BRENDA in 2013: integrated reactions, kinetic data, enzyme function data, improved disease classification: new options and contents in BRENDA. Nucleic Acids Research.

