# Supplementary material for: Annotation-based feature extraction from sets of SBML models
Source: J Biomed Semantics. 2015 Apr 15;6:20. doi: 10.1186/s13326-015-0014-4 (PMC4405863; doi:10.1186/s13326-015-0014-4)
Supplement: Supplementary file 1 — Supplementary material. A landscape table. This table lists for each of our seven test sets the contained models by their Biomodels Database ID. [file 13326_2015_14_MOESM1_ESM.pdf]

Table 1: List of models contained in the seven test sets: selection of published models from BioModels Database on the topics of the Cell Cycle, Ca-Oscillations, NFKB and Apoptosis; two random sets; and a set containing all curated models from Release 25 of BioModels Database. -All models were chosen from the curated branch of BioModels Database.

Cell Cycle (*CC*)	Ca-Oscillations (*CA*)	NFKB (*NFKB*)	Apoptosis (*APOP*)	Random Set 1 (*RS1*)	Random Set 2 (*RS2*)	BioModels Database (*BMDB*)
BIOMD0000000003	BIOMD0000000039	BIOMD0000000139	BIOMD0000000102	BIOMD0000000007	BIOMD0000000005	curated
BIOMD0000000005	BIOMD0000000043	BIOMD0000000140	BIOMD0000000103	BIOMD0000000015	BIOMD0000000010	branch
BIOMD0000000006	BIOMD0000000044	BIOMD0000000147	BIOMD0000000163	BIOMD0000000016	BIOMD0000000023	BioModels
BIOMD0000000007	BIOMD0000000045	BIOMD0000000226	BIOMD0000000220	BIOMD0000000029	BIOMD0000000048	Database
BIOMD0000000008	BIOMD0000000047	BIOMD0000000227	BIOMD0000000243	BIOMD0000000050	BIOMD0000000080	Release 25
BIOMD0000000056	BIOMD0000000058	BIOMD0000000230	BIOMD0000000252	BIOMD0000000061	BIOMD0000000081	(490 SBML files)
BIOMD0000000064	BIOMD0000000059	BIOMD0000000243	BIOMD0000000256	BIOMD0000000062	BIOMD0000000087	
BIOMD0000000069	BIOMD0000000114	BIOMD0000000288	BIOMD0000000407	BIOMD0000000075	BIOMD0000000105	
BIOMD0000000087	BIOMD0000000115	BIOMD0000000407	BIOMD0000000477	BIOMD0000000095	BIOMD0000000112	
BIOMD0000000107	BIOMD0000000117	BIOMD0000000451	BIOMD0000000523	BIOMD0000000107	BIOMD0000000118	
BIOMD0000000109	BIOMD0000000122	BIOMD0000000477	BIOMD0000000524	BIOMD0000000131	BIOMD0000000139	
BIOMD0000000110	BIOMD0000000145	BIOMD0000000489	BIOMD0000000525	BIOMD0000000173	BIOMD0000000141	
BIOMD0000000111	BIOMD0000000184		BIOMD0000000526	BIOMD0000000218	BIOMD0000000158	
BIOMD0000000144				BIOMD0000000225	BIOMD0000000168	
BIOMD0000000150				BIOMD0000000280	BIOMD0000000223	
BIOMD0000000168				BIOMD0000000290	BIOMD0000000254	
BIOMD0000000169				BIOMD0000000312	BIOMD0000000281	
BIOMD0000000181				BIOMD0000000314	BIOMD0000000282	
BIOMD0000000186				BIOMD0000000315	BIOMD0000000301	
BIOMD0000000187				BIOMD0000000324	BIOMD0000000313	
BIOMD0000000193				BIOMD0000000345	BIOMD0000000314	
BIOMD0000000194				BIOMD0000000361	BIOMD0000000315	
BIOMD0000000195				BIOMD0000000384	BIOMD0000000320	
BIOMD0000000196				BIOMD0000000391	BIOMD0000000335	
BIOMD0000000207				BIOMD0000000393	BIOMD0000000376	
BIOMD0000000208				BIOMD0000000425	BIOMD0000000391	
BIOMD0000000216				BIOMD0000000428	BIOMD0000000412	
BIOMD0000000228				BIOMD0000000433	BIOMD0000000418	
BIOMD0000000242				BIOMD0000000437	BIOMD0000000426	
BIOMD0000000265				BIOMD0000000455	BIOMD0000000433	
BIOMD0000000297				BIOMD0000000046	BIOMD0000000076	
BIOMD0000000318				BIOMD0000000117	BIOMD0000000253	
BIOMD0000000370				BIOMD0000000449	BIOMD0000000260	
BIOMD0000000409				BIOMD0000000471	BIOMD0000000429	
