# Supplementary material for: Annotation-based feature extraction from sets of SBML models
Source: J Biomed Semantics. 2015 Apr 15;6:20. doi: 10.1186/s13326-015-0014-4 (PMC4405863; doi:10.1186/s13326-015-0014-4)
Supplement: Supplementary file 2 — Depth of ontology entries. This file lists the number of annotations pointing to a certain depth within an ontology for each model set. [file 13326_2015_14_MOESM2_ESM.pdf]

BMDB

**Chebi**			**GO**			**SBO**		
depth	# of anno		depth	# of anno		depth	# of anno	
1	0	0	1	5	5	1	0	0
2	0	0	2	602	1204	2	40	80
3	22	66	3	882	2646	3	222	666
4	35	140	4	1081	4324	4	4248	16992
5	12	60	5	2046	10230	5	2769	13845
6	103	618	6	3128	18768	6	4515	27090
7	1344	9408	7	1661	11627	7	697	4879
8	548	4384	8	942	7536	8	517	4136
9	495	4455	9	261	2349	9	4	36
10	1026	10260	10	187	1870	10	0	0
11	382	4202	11	80	880	11	0	0
12	415	4980	12	7	84	12	0	0
13	1127	14651	13	0	0	13	0	0
14	47	658	14	0	0	14	0	0
15	167	2505	15	0	0	15	0	0
16	6	96	16	0	0	16	0	0
**avg**		**9,85913772**	**avg**		**5,653648226**	**avg**		**5,204734**
sum	5729			10882			13012	

CC

**Chebi**			**GO**			**SBO**		
depth	# of anno		depth	# of anno		depth	# of anno	
1	0	0	1	0	0	1	0	0
2	0	0	2	23	46	2	2	4
3	0	0	3	39	117	3	1	3
4	0	0	4	76	304	4	69	276
5	0	0	5	194	970	5	48	240
6	0	0	6	333	1998	6	147	882
7	0	0	7	168	1176	7	16	112
8	2	16	8	105	840	8	4	32
9	2	18	9	15	135	9	0	0
10	11	110	10	1	10	10	0	0
11	4	44	11	0	0	11	0	0
12	10	120	12	0	0	12	0	0
13	7	91	13	0	0	13	0	0
14	0	0	14	0	0	14	0	0
15	0	0	15	0	0	15	0	0
16	1	16	16	0	0	16	0	0
**avg**		**11,21621622**			**5,865828092**			**5,397213**
sum	37			954			287	

RS1

**Chebi**	**GO**	**SBO**

depth	# of anno		depth	# of anno		depth	# of anno	
4	1	4	2	27	54	2	7	14
5	10	50	3	20	60	3	6	18
6	12	72	4	53	212	4	422	1688
7	88	616	5	54	270	5	297	1485
8	59	472	6	125	750	6	431	2586
9	79	711	7	58	406	7	31	217
10	77	770	8	34	272	8	51	408
11	73	803	9	2	18			
12	64	768	10	3	30			
13	63	819	11	7	77			
14	52	728						
15	7	105						
**avg**		**10,11623932**			**5,610966057**			**5,153414**
sum	585			383			1245	

RS2

**Chebi**			**GO**			**SBO**		
depth	# of anno		depth	# of anno		depth	# of anno	
6	14	84	2	22	44	3	8	24
7	10	70	3	37	111	4	226	904
8	7	56	4	29	116	5	69	345
9	10	90	5	101	505	6	263	1578
10	56	560	6	333	1998	7	13	91
11	8	88	7	124	868	8	19	152
12	13	156	8	46	368			
13	29	377	9	16	144			
14	11	154	10	10	100			
15	2	30						
**avg**		**10,40625**			**5,924791086**			**5,173913**
sum	160			718			598	

APOP

**Chebi**			**GO**			**SBO**		
depth	# of anno		depth	# of anno		depth	# of anno	
3	12	36	2	22	44	2	2	4
10	1	10	3	47	141	4	85	340
13	1	13	4	58	232	5	74	370
			5	15	75	6	284	1704
			6	97	582	7	15	105
			7	5	35	8	36	288
			8	1	8			
			10	5	50			
**avg**		**4,214285714**			**4,668**			**5,667339**

sum	14			250			496	

NFKB								
**Chebi**			**GO**			**SBO**		
depth	# of anno		depth	# of anno		depth	# of anno	
-1	-1	0	2	5	10	2	1	2
			3	45	135	3	16	48
			4	41	164	4	60	240
			5	20	100	5	38	190
			6	141	846	6	118	708
			7	44	308	7	3	21
			8	10	80			
**avg**	**n/a**				**5,369281046**			**5,122881**
sum	-1			306			236	

CAOZ								
**Chebi**			**GO**			**SBO**		
depth	# of anno		depth	# of anno		depth	# of anno	
7	65	455	2	3	6	4	3	12
10	1	10	3	4	12	5	10	50
13	3	39	4	24	96	6	3	18
14	8	112	5	22	110			
			6	20	120			
			7	30	210			
			8	62	496			
			9	11	99			
			10	3	30			
			11	51	561			
			12	7	84			
**avg**		**8**			**7,696202532**			**5**
stdv								
sum	77			237			16	
