# Supplementary material for: Annotation-based feature extraction from sets of SBML models
Source: J Biomed Semantics. 2015 Apr 15;6:20. doi: 10.1186/s13326-015-0014-4 (PMC4405863; doi:10.1186/s13326-015-0014-4)
Supplement: Supplementary file 3 — Extracted features. This file lists extracted features and corresponding depth for each model set, feature size and ontology. [file 13326_2015_14_MOESM3_ESM.pdf]

Chebi	F15	M2 CC
CHEBI_16646	8	
CHEBI_24651	5	
CHEBI_25367	4	
CHEBI_25699	4	
CHEBI_25741	5	
CHEBI_26082	6	
CHEBI_33241	5	
CHEBI_33839	4	
CHEBI_35701	7	
CHEBI_36358	4	
CHEBI_36606	7	
CHEBI_51143	6	
CHEBI_63161	8	
CHEBI_63299	8	
CHEBI_64709	7	
**AVG**	**5,87**	

Chebi	F15	M4 CC
CHEBI_22563	4	
CHEBI_33608	5	
CHEBI_33694	5	
CHEBI_37096	13	
CHEBI_37787	9	
**AVG**	**7,20**	

Go	F15	M2 CC
GO_0003674	1	
GO_0005575	1	
GO_0006807	3	
GO_0009056	3	
GO_0009058	3	
GO_0040007	2	
GO_0044237	3	
GO_0044238	3	
GO_0044699	2	
GO_0050896	2	
GO_0051234	2	
GO_0065007	2	
GO_0071704	3	
GO_0071840	2	
**AVG**	**2,29**	

Go	F15	M4 CC
GO_0000216	2	
GO_0004693	8	
GO_0005575	1	
GO_0022411	4	
GO_0030163	5	
GO_0032268	6	
GO_0045750	2	
GO_0051726	5	
GO_0065009	3	
GO_0071822	5	
**AVG**	**4,10**	

SBO	F15	M2 CC
SBO_0000009	4	
SBO_0000177	6	
SBO_0000179	6	
SBO_0000180	6	
SBO_0000181	6	
SBO_0000182	6	
SBO_0000205	4	
SBO_0000245	4	
SBO_0000253	4	
SBO_0000290	4	
SBO_0000291	4	
SBO_0000308	4	
SBO_0000342	4	
SBO_0000360	4	
SBO_0000374	3	
**AVG**	**4,60**	

SBO	F15	M4 CC
SBO_0000009	4	
SBO_0000231	2	
SBO_0000252	6	
SBO_0000336	4	
**AVG**	**4,00**	

Chebi	F5	M2 CC
CHEBI_33285	7	
CHEBI_33302	5	
CHEBI_33304	5	
CHEBI_35701	7	
CHEBI_36357	3	
**AVG**	**5,40**	

Chebi	F5	M4 CC
CHEBI_22563	4	
CHEBI_33608	5	
CHEBI_33694	5	
CHEBI_37096	13	
CHEBI_37787	9	
**AVG**	**7,20**	

Go	F5	M2 CC
GO_0008152	2	
GO_0009987	2	
GO_0044699	2	
GO_0065007	2	
GO_0071840	2	
**AVG**	**2,00**	

Go	F5	M4 CC
GO_0022411	4	
GO_0030163	5	
GO_0051726	5	
GO_0065009	3	
GO_0071822	5	
**AVG**	**4,40**	

SBO	F5	M2 CC
SBO_0000003	2	
SBO_0000236	2	
SBO_0000374	3	
SBO_0000375	3	
SBO_0000545	2	
**AVG**	**2,40**	

SBO	F5	M4 CC
SBO_0000009	4	
SBO_0000231	2	
SBO_0000252	6	
SBO_0000336	4	
**AVG**	**4,00**	

Chebi	F15	M2 F
CHEBI_18059	7	
CHEBI_24835	3	
CHEBI_24870	3	
CHEBI_25367	4	
CHEBI_26082	6	
CHEBI_33241	5	
CHEBI_33259	3	
CHEBI_33285	7	
CHEBI_33304	5	
CHEBI_33674	4	
CHEBI_33839	4	
CHEBI_35701	7	
CHEBI_50906	1	
CHEBI_51143	6	
CHEBI_64709	7	
**AVG**	**4,80**	

Chebi	F15	M4 F
CHEBI_24835	3	
CHEBI_24870	3	
CHEBI_26082	6	
CHEBI_33241	5	
CHEBI_33259	3	
CHEBI_33636	6	
CHEBI_33695	6	
CHEBI_35155	7	
CHEBI_35569	7	
CHEBI_35701	7	
CHEBI_47019	11	
CHEBI_61120	7	
CHEBI_63161	8	
CHEBI_63367	9	
CHEBI_64709	7	
**AVG**	**6,33**	

Go	F15	M2 F
GO_0003674	1	
GO_0005575	1	
GO_0008152	2	
GO_0009987	2	
GO_0032501	2	
GO_0032502	2	
GO_0040007	2	
GO_0044699	2	
GO_0048511	2	
GO_0050896	2	
GO_0051234	2	
GO_0051704	2	
GO_0065007	2	
GO_0071840	2	
**AVG**	**1,86**	

Go	F15	M4 F
GO_0003674	1	
GO_0005575	1	
GO_0009987	2	
GO_0043170	4	
GO_0071822	5	
**AVG**	**2,60**	

SBO	F15	M2 F
SBO_0000003	2	
SBO_0000064	2	
SBO_0000241	3	
SBO_0000245	4	
SBO_0000247	4	
SBO_0000253	4	
SBO_0000285	4	
SBO_0000290	4	
SBO_0000291	4	
SBO_0000374	3	
SBO_0000375	3	
SBO_0000405	4	
SBO_0000409	3	
SBO_0000412	3	
SBO_0000545	2	
**AVG**	**3,27**	

SBO	F15	M4 F
SBO_0000003	2	
SBO_0000009	4	
SBO_0000064	2	
SBO_0000167	4	
SBO_0000240	3	
**AVG**	**3,00**	

Chebi	F5	M2 F
CHEBI_24870	3	
CHEBI_33302	5	
CHEBI_33304	5	
CHEBI_33582	5	
CHEBI_36357	3	
**AVG**	**4,20**	

Chebi	F5	M4 F
CHEBI_24870	3	
CHEBI_26082	6	
CHEBI_33241	5	
CHEBI_33695	6	
CHEBI_61120	7	
**AVG**	**5,40**	

Go	F5	M2 F
GO_0003674	1	
GO_0008152	2	
GO_0009987	2	
GO_0044699	2	
GO_0065007	2	
**AVG**	**1,80**	

Go	F5	M4 F
GO_0003674	1	
GO_0005575	1	
GO_0009987	2	
GO_0043170	4	
GO_0071822	5	
**AVG**	**2,60**	

SBO	F5	M2 F
SBO_0000003	2	
SBO_0000064	2	
SBO_0000231	2	
SBO_0000236	2	
SBO_0000545	2	
**AVG**	**2,00**	

SBO	F5	M4 F
SBO_0000003	2	
SBO_0000009	4	
SBO_0000064	2	
SBO_0000167	4	
SBO_0000240	3	
**AVG**	**3,00**	

Chebi	F15	M2 R1
CHEBI_18059	7	
CHEBI_24835	3	
CHEBI_24870	3	
CHEBI_25367	4	
CHEBI_25806	6	
CHEBI_26082	6	
CHEBI_26835	6	
CHEBI_33241	5	
CHEBI_33259	3	
CHEBI_33285	7	
CHEBI_33674	4	
CHEBI_33694	5	
CHEBI_35701	7	
CHEBI_51143	6	
CHEBI_64709	7	
**AVG**	**5,27**	

Chebi	F15	M4 R1
CHEBI_22563	4	
CHEBI_24835	3	
CHEBI_25741	5	
CHEBI_26082	6	
CHEBI_33241	5	
CHEBI_33252	3	
CHEBI_33259	3	
CHEBI_33608	5	
CHEBI_33695	6	
CHEBI_35701	7	
CHEBI_61120	7	
CHEBI_63367	9	
CHEBI_64709	7	
**AVG**	**5,38**	

Go	F15	M2 R1
GO_0003674	1	
GO_0005575	1	
GO_0006807	3	
GO_0009056	3	
GO_0009058	3	
GO_0044237	3	
GO_0044238	3	
GO_0044699	2	
GO_0044710	3	
GO_0048511	2	
GO_0050896	2	
GO_0051234	2	
GO_0065007	2	
GO_0071704	3	
GO_0071840	2	
**AVG**	**2,33**	

Go	F15	M4 R1
GO_0003674	1	
GO_0005575	1	
GO_0006810	3	
GO_0009987	2	
GO_0016088	2	
GO_0043170	4	
GO_0045750	2	
**AVG**	**2,14**	

SBO	F15	M2 R1
SBO_0000064	2	
SBO_0000177	6	
SBO_0000179	6	
SBO_0000180	6	
SBO_0000182	6	
SBO_0000185	5	
SBO_0000205	4	
SBO_0000241	3	
SBO_0000247	4	
SBO_0000250	6	
SBO_0000253	4	
SBO_0000285	4	
SBO_0000290	4	
SBO_0000377	6	
SBO_0000545	2	
**AVG**	**4,53**	

SBO	F15	M4 R1
SBO_0000009	4	
SBO_0000064	2	
SBO_0000176	5	
SBO_0000252	6	
**AVG**	**4,25**	

Chebi	F5	M2 R1
CHEBI_24870	3	
CHEBI_33302	5	
CHEBI_33304	5	
CHEBI_33582	5	
CHEBI_36357	3	
**AVG**	**4,20**	

Chebi	F5	M4 R1
CHEBI_22563	4	
CHEBI_26082	6	
CHEBI_33241	5	
CHEBI_33695	6	
CHEBI_61120	7	
**AVG**	**5,60**	

Go	F5	M2 R1
GO_0003674	1	
GO_0008152	2	
GO_0009987	2	
GO_0044699	2	
GO_0051234	2	
**AVG**	**1,80**	

Go	F5	M4 R1
GO_0003674	1	
GO_0005575	1	
GO_0006810	3	
GO_0009987	2	
GO_0043170	4	
**AVG**	**2,20**	

SBO	F5	M2 R1
SBO_0000545	2	
SBO_0000064	2	
SBO_0000231	2	
SBO_0000240	3	
SBO_0000241	3	
**AVG**	**2,40**	

SBO	F5	M4 R1
SBO_0000009	4	
SBO_0000064	2	
SBO_0000176	5	
SBO_0000252	6	
**AVG**	**4,25**	

Chebi	F15	M2 R2
CHEBI_24835	3	
CHEBI_24870	3	
CHEBI_25806	6	
CHEBI_26082	6	
CHEBI_26835	6	
CHEBI_33259	3	
CHEBI_33674	4	
CHEBI_33694	5	
CHEBI_35701	7	
CHEBI_36962	6	
CHEBI_37577	4	
CHEBI_50906	1	
CHEBI_51143	6	
CHEBI_64709	7	
CHEBI_72695	5	
**AVG**	**4,80**	

Chebi	F15	M4 R2
CHEBI_26816	9	
CHEBI_30412	6	
CHEBI_33636	6	
CHEBI_33674	4	
CHEBI_33699	10	
CHEBI_37848	5	
CHEBI_60240	7	
CHEBI_63367	9	
CHEBI_64709	7	
**AVG**	**7,00**	

Go	F15	M2 R2
GO_0003674	1	
GO_0005575	1	
GO_0008152	2	
GO_0009987	2	
GO_0032501	2	
GO_0032502	2	
GO_0040011	2	
GO_0044699	2	
GO_0048511	2	
GO_0050896	2	
GO_0051234	2	
GO_0051704	2	
GO_0065007	2	
GO_0071840	2	
**AVG**	**1,86**	

Go	F15	M4 R2
GO_0003674	1	
GO_0003675	2	
GO_0005623	2	
GO_0022411	4	
GO_0043170	4	
GO_0044238	3	
GO_0071822	5	

SBO	F15	M2 R2
SBO_0000003	2	
SBO_0000009	4	
SBO_0000241	3	
SBO_0000245	4	
SBO_0000247	4	
SBO_0000253	4	
SBO_0000257	4	
SBO_0000258	4	
SBO_0000259	4	
SBO_0000290	4	
SBO_0000291	4	
SBO_0000308	4	
SBO_0000374	3	
SBO_0000375	3	
SBO_0000380	4	
**AVG**	**3,67**	

SBO	F15	M4 R2
SBO_0000009	4	
SBO_0000167	4	
SBO_0000240	3	
SBO_0000289	3	
**AVG**	**3,50**	

Chebi	F5	M2 R2
CHEBI_33302	5	
CHEBI_33304	5	
CHEBI_33582	5	
CHEBI_33674	4	
CHEBI_36357	3	
**AVG**	**4,40**	

Chebi	F5	M4 R2
CHEBI_26816	9	
CHEBI_30412	6	
CHEBI_33699	10	
CHEBI_60240	7	
CHEBI_63367	9	
**AVG**	**8,20**	

Go	F5	M2 R2
GO_0003674	1	
GO_0008152	2	
GO_0009987	2	
GO_0044699	2	
GO_0071840	2	
**AVG**	**1,80**	

Go	F5	M4 R2
GO_0005623	2	
GO_0022411	4	
GO_0043170	4	
GO_0044238	3	
GO_0071822	5	
**AVG**	**3,60**	

SBO	F5	M2 R2
SBO_0000003	2	
SBO_0000231	2	
SBO_0000240	3	
SBO_0000241	3	
SBO_0000545	2	
**AVG**	**2,40**	

SBO	F5	M4 R2
SBO_0000009	4	
SBO_0000167	4	
SBO_0000240	3	
SBO_0000289	3	
**AVG**	**3,50**	

Go	F15	M2 Apop
GO_0003824	2	
GO_0005488	2	
GO_0005575	1	
GO_0009056	3	
GO_0009987	2	
GO_0030234	2	
GO_0032501	2	
GO_0044238	3	
GO_0044699	2	
GO_0050896	2	
GO_0051234	2	
GO_0065007	2	
GO_0071704	3	
GO_0071840	2	
**AVG**	**2,14**	

Go	F15	M4 Apop
GO_0002090	6	
GO_0005515	3	
GO_0016265	3	
GO_0030693	2	
GO_0031264	4	
GO_0043027	4	
GO_0044257	6	
GO_0065003	5	
GO_0071822	5	
**AVG**	**4,22**	

Go	F5	M2 Apop
GO_0003674	1	
GO_0005575	1	
GO_0008152	2	
GO_0009987	2	
GO_0071840	2	
**AVG**	**1,60**	

Go	F5	M4 Apop
GO_0005515	3	
GO_0030693	2	
GO_0044257	6	
GO_0065003	5	
GO_0071822	5	
**AVG**	**4,20**	

Go	F15	M2 NFKB
GO_0003674	1	
GO_0005575	1	
GO_0006807	3	
GO_0009056	3	
GO_0009058	3	
GO_0044237	3	
GO_0044238	3	
GO_0044699	2	
GO_0044710	3	
GO_0050896	2	
GO_0051234	2	
GO_0065007	2	
GO_0071704	3	
GO_0071840	2	
**AVG**	**2,36**	

Go	F15	M4 NFKB
GO_0005515	3	
GO_0005634	5	
GO_0006886	5	
GO_0016563	2	
GO_0022607	4	
GO_0044257	6	
GO_0071822	5	

Go	F5	M2 NFKB
GO_0003674	1	
GO_0008152	2	
GO_0009987	2	
GO_0044699	2	
GO_0071840	2	
**AVG**	**1,80**	

Go	F5	M4 NFKB
GO_0005515	3	
GO_0006886	5	
GO_0022607	4	
GO_0044257	6	
GO_0071822	5	
**AVG**	**4,60**	

Go	F15	M2 CaOz
GO_0003824	2	
GO_0004872	2	
GO_0005215	2	
GO_0005488	2	
GO_0005575	1	
GO_0007204	11	
GO_0022411	4	
GO_0032469	10	
GO_0044237	3	
GO_0050789	3	
GO_0051234	2	
GO_0051481	11	
GO_0051716	3	
GO_0060089	2	
GO_0065009	3	
**AVG**	**4,07**	

Go	F15	M4 CaOz
GO_0005217	9	
GO_0005783	5	
GO_0005829	5	
GO_0006816	8	
GO_0015085	9	
GO_0017111	7	
GO_0038023	3	
GO_0051480	10	
**AVG**	**7,00**	

Go	F5	M2 CaOz
GO_0003674	1	
GO_0009987	2	
GO_0044699	2	
GO_0051234	2	
GO_0065007	2	
**AVG**	**1,80**	

Go	F5	M4 CaOz
GO_0005217	9	
GO_0005829	5	
GO_0006816	8	
GO_0015085	9	
GO_0051480	10	
**AVG**	**8,20**	
